# HADC5 deacetylates MKL1 to dampen TNF-α induced pro-inflammatory gene transcription in macrophages

**DOI:** 10.18632/oncotarget.21670

**Published:** 2017-10-09

**Authors:** Zilong Li, Hao Qin, Jianfei Li, Liming Yu, Yuyu Yang, Yong Xu

**Affiliations:** ^1^ Key Laboratory of Targeted Intervention of Cardiovascular Disease, Innovative Collaboration Center for Cardiovascular Translational Medicine, Department of Pathophysiology, Nanjing Medical University, Nanjing, China; ^2^ State Key Laboratory of Natural Medicines, China Pharmaceutical University, Nanjing, China

**Keywords:** transcriptional regulation, MKL1, HDAC5, post-translational modification, deacetylation

## Abstract

Macrophage-dependent inflammatory response on the one hand functions as a key line of defense in host immunity but on the other hand underlies the pathogenesis of a host of human pathologies when aberrantly activated. Our previous investigations have led to the identification of megakaryocytic leukemia 1 (MKL1) as a key co-factor of NF-κB/p65 participating in TNF-α induced pro-inflammatory transcription in macrophages. How post-translational modifications contribute to the modulation of MKL1 activity remains an underexplored subject matter. Here we report that the lysine deacetylase HDAC5 interacts with and deacetylates MKL1 in cells. TNF-α treatment down-regulates HDAC5 expression and expels HDAC5 from the promoters of pro-inflammatory genes in macrophages. In contrast, over-expression of HDAC5 attenuates TNF-α induced pro-inflammatory transcription. Mechanistically, HDAC5-mediated MKL1 deacetylation disrupts the interaction between MKL1 and p65. In addition, deacetylation of MKL1 by HDAC5 blocks its nuclear translocation in response to TNF-α treatment. In conclusion, our work has identified an important pathway that contributes to the regulation of pro-inflammatory response in macrophages.

## INTRODUCTION

A key difference between prokaryotes and eukaryotes is the presence of a mature and full-fledged post-translational modification (PTM) machinery in the latter organisms [1]. A diverse range of chemical groups can be covalently attached to different amino acid residues of translated proteins to alter their binding partners, sub-cellular localizations, turnover rates, and, specifically for transcription factors (TFs), selective occupancies on target DNA [2]. Modulation of TF activity via PTM represents a paradigm in transcriptional regulation. The master regulator of pro-inflammatory transcription NF-κB and other components of the NF-κB signaling pathway can be modified by phosphorylation, acetylation, SUMOylation, ubiquitination, and methylation [3]. Acetylation, for instance, can be catalyzed by lysine acetyltransferases (KATs) and lysine deacetylases (KDACs) [4]. Both the p50 subunit and the p65 subunit of NF-κB can be acetylated although the responsible KATs appear to be different depending on the context [5, 6]. Similarly, several different KDACs including SIRT1 [7], SIRT2 [8], HDAC1 [9], and HDAC6 [10] are known to possess deacetylase activities towards NF-κB.

The outcome of NF-κB mediated pro-inflammatory transcription is influenced by its many co-factors. Our previous work has led to the identification of megakaryocytic leukemia 1 (MKL1), variably termed myocardin-related transcription factor A (MRTF-A), as a co-factor for p65 in vascular endothelial cells [11, 12]. MKL1 is universally present in all mammalian tissues and cells [13] and subsequent studies have found that MKL1 can modulate p65-dependent pro-inflammatory transcription in macrophages [14]. The primary mechanism whereby MKL1 enhances p65 activity is attributable to the fact that MKL1 interacts with and recruits a histone H4K4 methyltransferase complex (COMPASS) to the promoters of NF-κB target genes [15, 16]. In the realm of PTM, MKL1 is not a stranger. Nakagawa and Kuzumaki have reported that MKL1 is SUMOylated in HEK293 cells and that SUMOylation of MKL1 represses its activity without altering its nuclear enrichment or half-life [17]. Several independent investigations have documented the dynamic modulation of MKL1 phosphorylation in HeLa cells [18], smooth muscle cells [19], and macrophages [20] with varying consequences. Wang and colleagues have recently shown that myocardin, a protein structurally and functionally related to MKL1, can be acetylated *in vivo* [21]. Building on this discovery, we approached the question as to whether the ability of MKL1 to regulate pro-inflammatory transcription in macrophages might be calibrated by its acetylation status. We have found that p300/CBP associated factor (PCAF) acetylates MKL1 to enhance the transcription of pro-inflammatory mediators in macrophages [22]. We report here that histone deacetylase 5 (HDAC5) interacts with and deacetylates MKL1 to dampen TNF-α induced pro-inflammatory transcription in macrophages.

## RESULTS

### HDAC5 interacts with MKL1 and deacetylates MKL1

**Figure 1:**
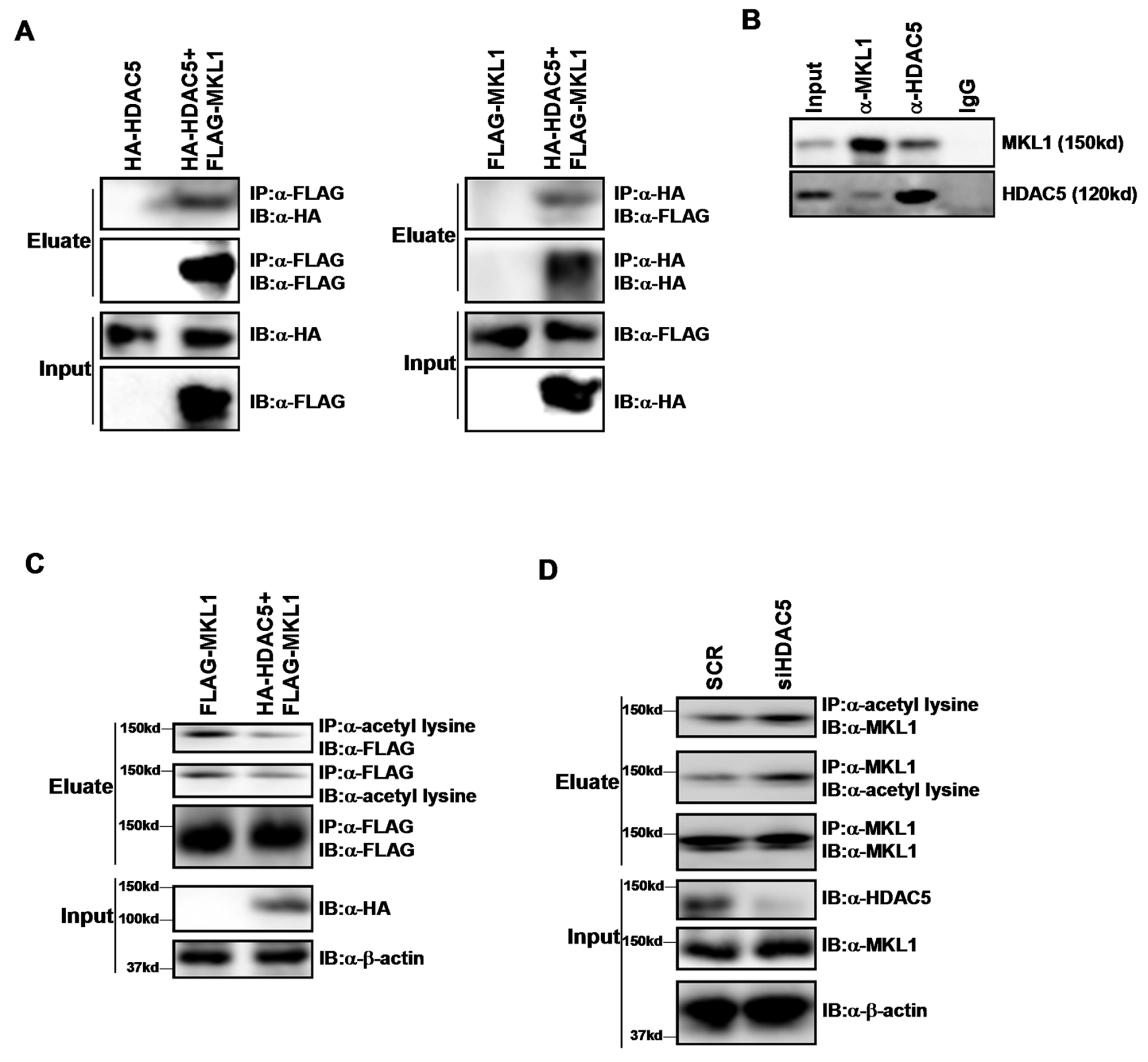
HDAC5 interacts with MKL1 and deacetylates MKL1 **(A)** HA-tagged HDAC5 and FLAG-tagged MKL1 were transfected into HEK293 cells. Immunoprecipitation assay was performed with indicated antibodies. **(B)** Whole cell lysates were isolated from THP-1 cells and immunoprecipitated with indicated antibodies. **(C)** FLAG-tagged MKL1 were transfected into HEK293 cells with or without HDAC5. Immunoprecipitation assay was performed with indicated antibodies. **(D)** THP-1 cells were transfected with siRNA targeting HDAC5 or scrambled siRNA (SCR). Immunoprecipitation assay was performed with indicated antibodies. All experiments have been repeated three times.

Cao *et al* have previously reported that Myocardin, a transcriptional modulator closely related to MKL1, is deacetylated by HDAC5 in smooth muscle cells [21]. Therefore, we hypothesized that HDAC5 might function as a deacetylase for MKL1 as well. To examine this potential MKL1-HDAC5 interplay, we performed co-immunoprecipitation (Co-IP) experiments. FLAG-tagged MKL1 expression construct and HA-tagged HDAC5 expression construct were co-transfected into HEK293 cells. An anti-FLAG antibody precipitated MKL1 while at the same time pulled down HDAC5 (Figure 1A, left panel). Reciprocal Co-IP demonstrated that an anti-HA antibody simultaneously brought down HDAC5 and MKL1 (Figure 1A, right panel). More important, endogenous MKL1 and HDAC5 also formed a complex as evidenced by Co-IP experiments performed with whole cell lysates extracted from THP-1 cells (Figure 1B).

Immunoprecipitation with an anti-acetyl lysine antibody revealed that MKL1 was acetylated in cells; over-expression of HDAC5 attenuated MKL1 acetylation levels (Figure 1C). In contrast, depletion of HDAC5 by siRNA enhanced MKL1 acetylation in THP-1 cells (Figure 1D) and RAW264 cells (Supplementary Figure 1). Together, these data suggest that HDAC5 interacts with MKL1 and deacetylates MKL1.

### TNF-α represses HDAC5 expression and reduces the nuclear level of HDAC5 in macrophages

**Figure 2:**
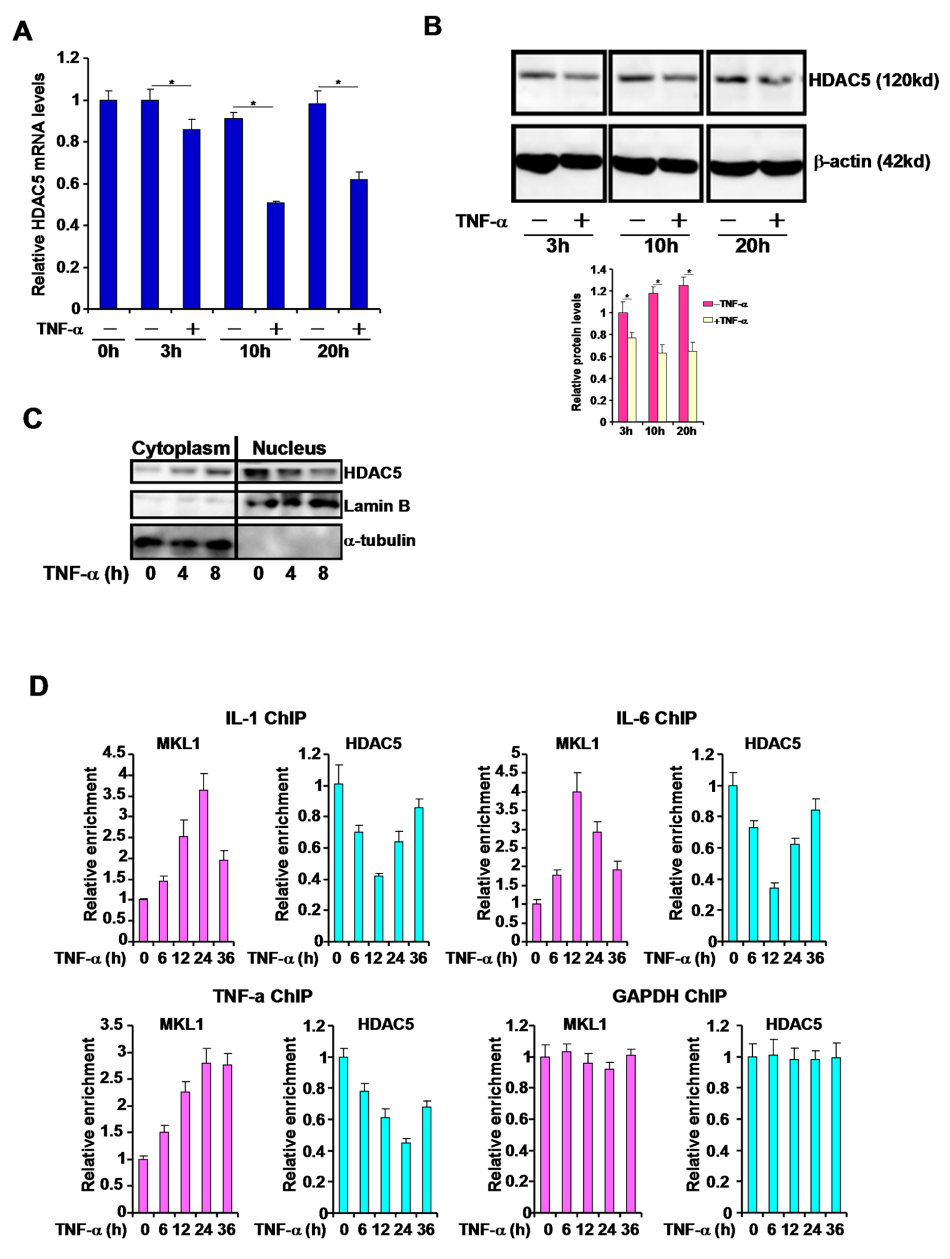
TNF-α represses HDAC5 expression and reduces the nuclear level of HDAC5 in macrophages **(A, B)** THP-1 cells were treated with TNF-α (10ng/ml) and harvested at indicated time points. Expression levels of HDAC5 were assessed by qPCR (A) and Western (B). Nuclear levels of HDAC5 were examined by cell fractionation followed immunoblotting **(C)**. ChIP assays were performed with anti-HDAC5 or anti-MKL1 **(D)**. All experiments have been repeated three times.

We have previously shown that MKL1 can be activated by the pro-inflammatory cytokine TNF-α in macrophages [15, 16]. We then sought to determine the effects of TNF-α on HDAC5. Stimulation of THP-1 cells with TNF-α resulted in a small but significant decrease in HDAC5 mRNA expression as early as 3 hours after treatment. Prolonged treatment led to further decreases of HDAC5 levels at 10 hours and 20 hours (Figure 2A). Western blotting analyses showed that HDAC5 protein expression was equally down-regulated by TNF-α treatment (Figure 2B). Furthermore, TNF-α stimulation appeared to reduce the nuclear level of HDAC5 as evidenced by cell fractionation experiment (Figure 2C). Finally, chromatin immunoprecipitation (ChIP) assay showed that occupancies of HDAC5 on the promoter regions of pro-inflammatory genes were significantly attenuated by TNF-α treatment (Figure 2D). Collectively, these data suggest that HDAC5 expression and activity may be negatively regulated by TNF-α in macrophages.

### HDAC5 represses MKL1 mediated pro-inflammatory transcription

**Figure 3:**
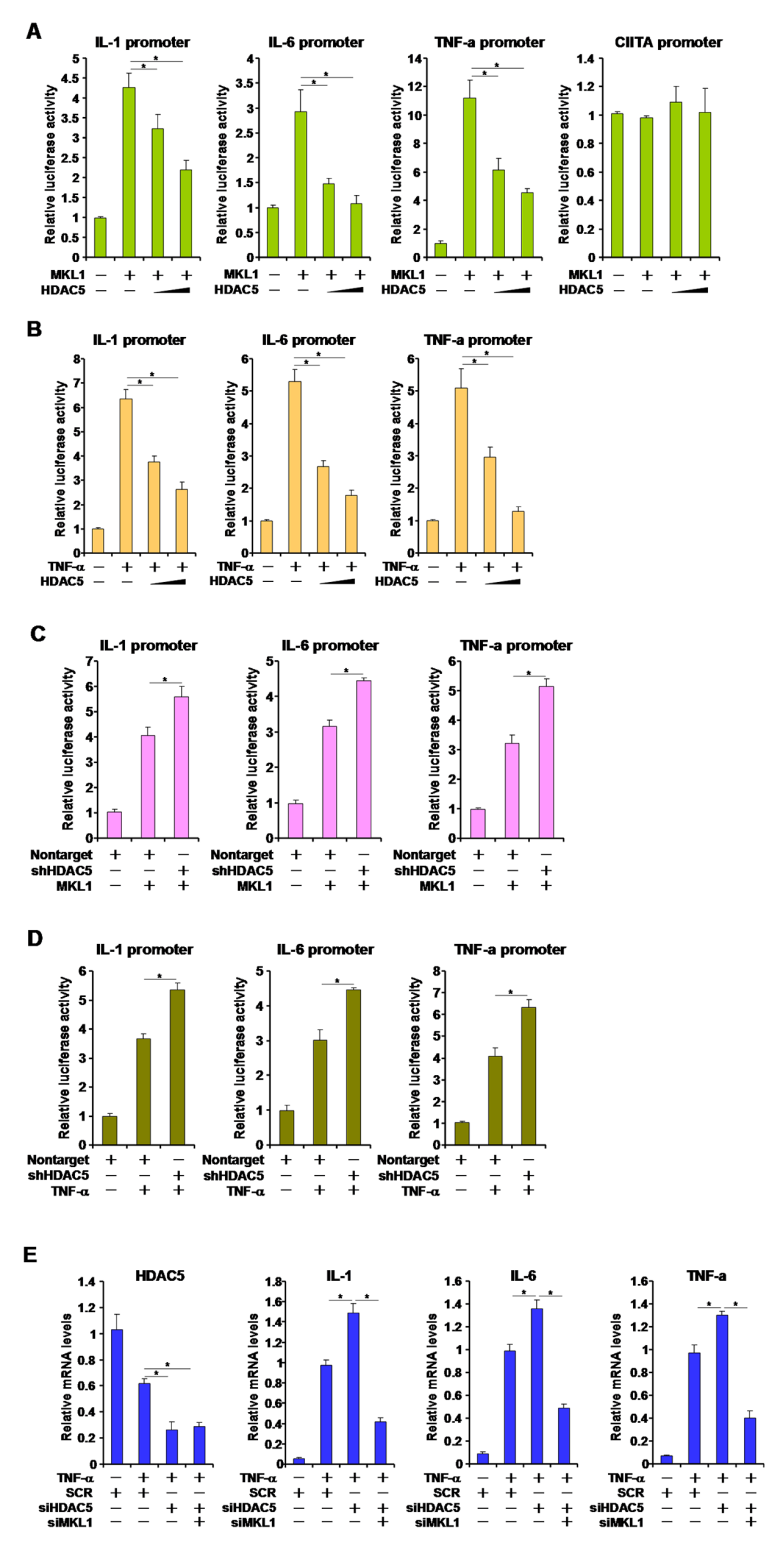
HDAC5 represses MKL1 mediated pro-inflammatory transcription **(A)** Different promoter constructs were transfected into HEK293 cells with MKL1 and/or HDAC5 expression construct. Luciferase activities were normalize by protein concentration and GFP fluorescence. **(B)** Different promoter constructs were transfected into HEK293 cells with HDAC5 expression construct followed by treatment with TNF-α (10ng/ml) for 12 hours. Luciferase activities were normalize by protein concentration and GFP fluorescence. **(C)** Different promoter constructs were transfected into RAW264 cells with MKL1 and/or HDAC5 shRNA construct. Luciferase activities were normalize by protein concentration and GFP fluorescence. **(D)** Different promoter constructs were transfected into RAW264 cells with HDAC5 shRNA construct followed by treatment with TNF-α (10ng/ml) for 12 hours. Luciferase activities were normalize by protein concentration and GFP fluorescence. **(E)** THP-1 cells were transfected with siRNA targeting HDAC5 or scrambled siRNA (SCR) followed by treatment with TNF-α (10ng/ml) for 12 hours. Expression of pro-inflammatory mediators was measured by qPCR. All experiments have been repeated three times.

It has been previously demonstrated that MKL1 mediates the trans-activation of pro-inflammatory mediators by TNF-α in macrophages [15, 16]. We asked whether HDAC5 could contribute to TNF-α-induced and MKL1-dependent trans-activation of pro-inflammatory genes. Over-expression of MKL1 stimulated the activities of pro-inflammatory gene promoters including IL-1, IL-6, and TNF-α in reporter assays. HDAC5, however, antagonized the up-regulation of promoter activities in a dose-dependent manner (Figure 3A). MKL1 did not, however, alter the promoter activity of class II transactivator (CIITA) either with or without HDAC5. We also observed that HDAC5 was able to reverse TNF-α induced trans-activation of pro-inflammatory gene promoters in a similar fashion (Figure 3B). We have previously shown that four lysine residues within the N-terminus of MKL1 is acetylated by PCAF [22]. An acetylation defective mutant of MKL1, while being less potent in trans-activating the promoters of pro-inflammatory genes compared to wild type MKL1, was resistant to HDAC5 over-expression, suggesting that HDAC5 might target the same set of lysine residues as PCAF (Supplementary Figure 2).

On the other hand, short hairpin (shRNA) mediated HDAC5 silencing further potentiated the trans-activation of pro-inflammatory gene promoters by both MKL1 (Figure 3C) and TNF-α (Figure 3D). Similarly, depletion of endogenous HDAC5 by small interfering RNA (siRNA) also enhanced the up-regulation of pro-inflammatory mediator messages by TNF-α in THP-1 cells, which was pre-empted by simultaneous knockdown of MKL1 suggesting that HDAC5 functions through MKL1 (Figure 3E). Together, these data suggest that HDAC5 represses pro-inflammatory transcription likely through modulating MKL1 activity.

### HDAC5 dampens the recruitment of MKL1 to pro-inflammatory gene promoters by altering its nuclear accumulation

**Figure 4:**
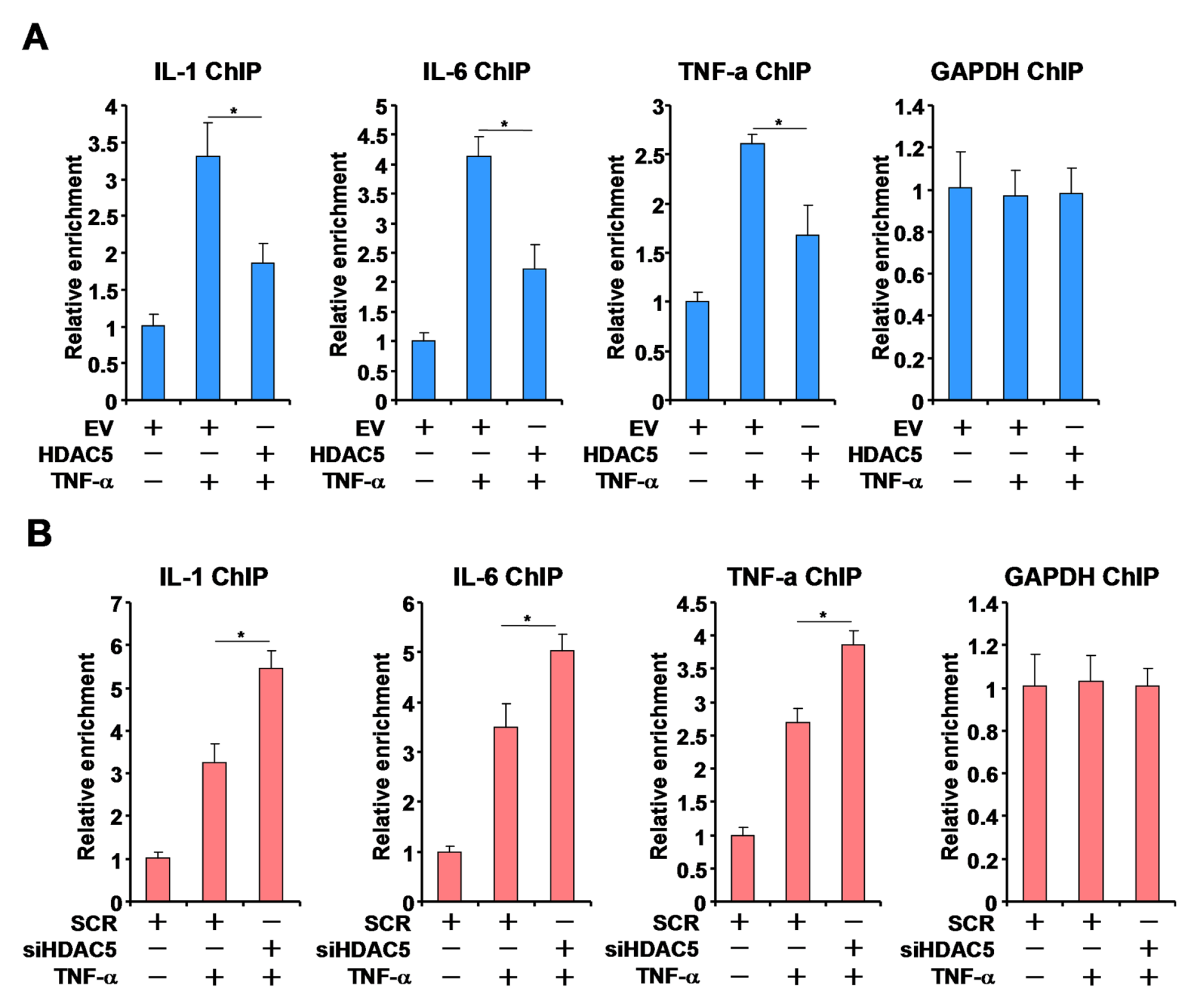
HDAC5 alters the recruitment of MKL1 to pro-inflammatory gene promoters **(A)** THP-1 cells were transfected with HDAC5 expression construct or an empty vector (EV) followed by treatment with TNF-α (10ng/ml) for 12 hours. ChIP assays were performed with anti-MKL1. **(B)** THP-1 cells were transfected with siRNA targeting HDAC5 or scrambled siRNA (SCR) followed by treatment with TNF-α (10ng/ml) for 12 hours. ChIP assays were performed with anti-MKL1. All experiments have been repeated three times.

We next investigated the potential mechanism(s) whereby HDAC5 may regulate MKL1 activity. ChIP assay showed that in response to TNF-α stimulation, MKL1 occupancies on the pro-inflammatory gene promoters were significantly up-regulated. HDAC5 over-expression overcame this trend by attenuating the binding of MKL1 (Figure 4A). Conversely, HDAC5 knockdown significantly enhanced the recruitment of MKL1 to the gene promoters (Figure 4B).

**Figure 5:**
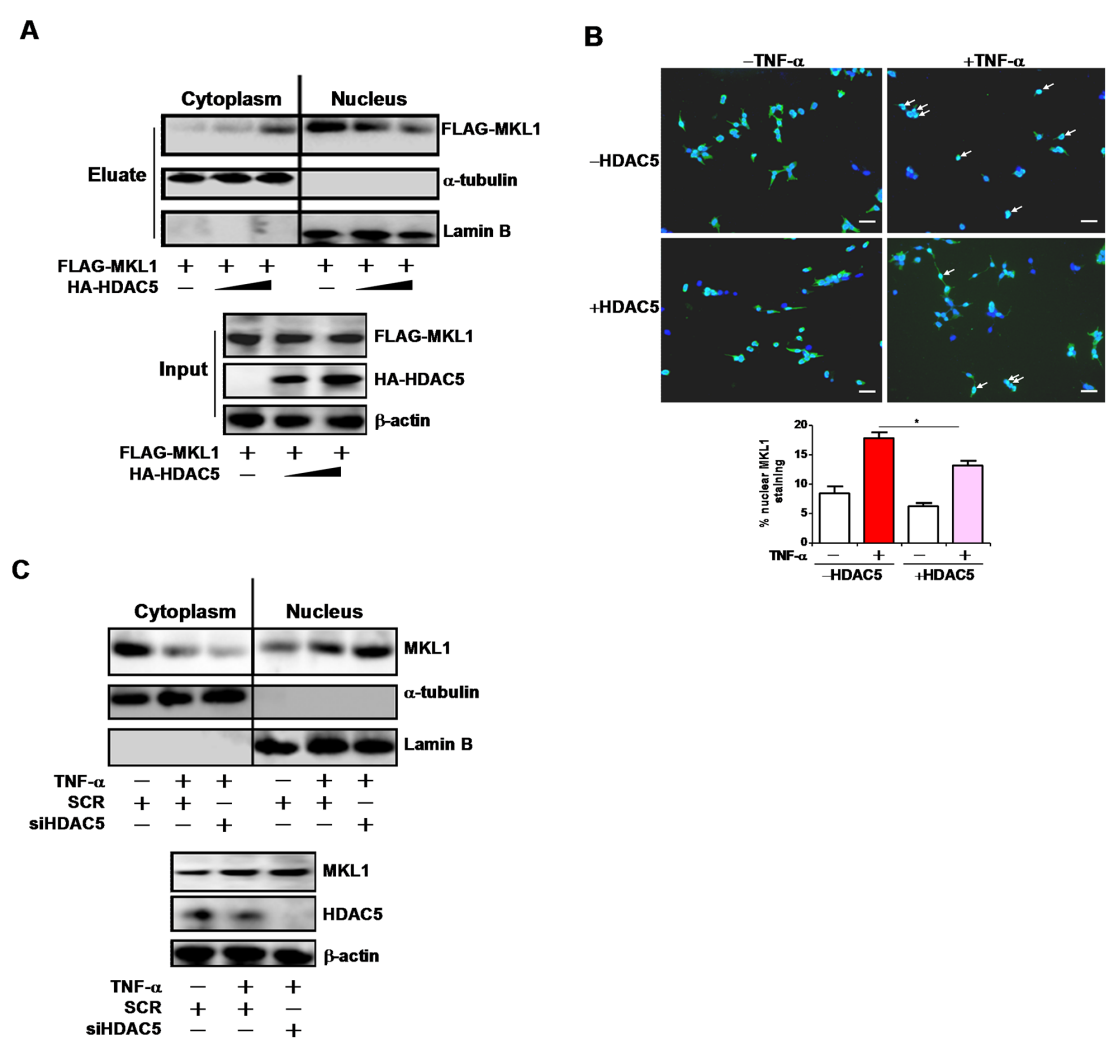
HDAC5 blocks MKL1 nuclear translocation **(A)** FLAG-tagged MKL1 was transfected into HEK293 cells in the presence or absence of HDAC5. Cells were fractionated and Western blotting was performed with indicated antibodies. **(B)** FLAG-tagged MKL1 was transfected into HEK293 cells in the presence or absence of HDAC5 with TNF-α (10ng/ml) for 6 hours. Sub-cellular localization of MKL1 was evaluated by immunoprecipitation using an anti-FLAG antibody. Nuclear MKL1 staining was quantified by Image J and expressed as percentage of overall MKL1 staining. Scale bar, 25μm. **(C)** THP-1 cells were transfected with siRNA targeting HDAC5 or scrambled siRNA (SCR) followed by treatment with TNF-α (10ng/ml) for 12 hours. Cells were fractionated and Western blotting was performed with indicated antibodies. All experiments have been repeated three times.

MKL1 can shuttle between the cytoplasm and the nucleus [15]. We therefore asked whether HDAC5 could alter MKL1 nuclear enrichment. Over-expression of HDAC5 disrupted nuclear translocation of MKL1 as evidenced by both cell fractionation/Western blotting (Figure 5A) and immunofluorescence staining (Figure 5B). On the contrary, HDAC5 knockdown resulted in augmented nuclear accumulation of MKL1 in response to TNF-α treatment (Figure 5C). Based on these observations, we conclude that HDAC5 may weaken the binding of MKL1 to target promoters by limiting its nuclear accumulation.

### HDAC5 disrupts the interaction between MKL1 and NF-κB

**Figure 6:**
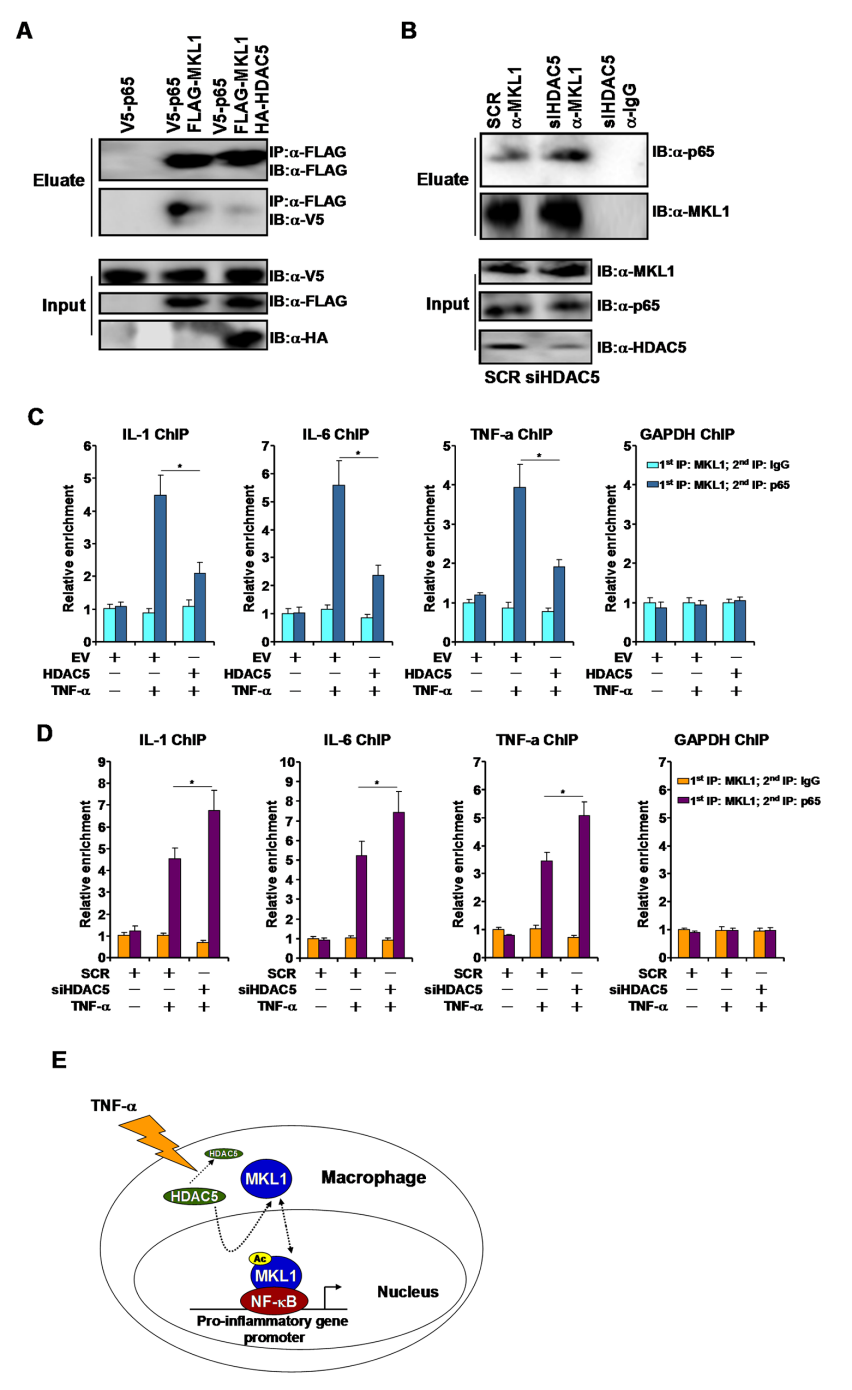
HDAC5 disrupts the interaction between MKL1 and p65 **(A)** FLAG-tagged MKL1 and V5-tagged p65 were transfected into HEK293 cells in the presence or absence of HDAC5. Immunoprecipitation was performed with anti-FLAG. **(B)** THP-1 cells were transfected with siRNA targeting HDAC5 or scrambled siRNA (SCR) followed by treatment with TNF-α (10ng/ml). Immunoprecipitation was performed with anti-MKL1. **(C)** THP-1 cells were transfected with HDAC5 expression construct or an empty vector (EV) followed by treatment with TNF-α (10ng/ml). Re-ChIP assays were performed with indicated antibodies. **(D)** THP-1 cells were transfected with siRNA targeting HDAC5 or scrambled siRNA (SCR) followed by treatment with TNF-α (10ng/ml). Re-ChIP assays were performed with indicated antibodies. **(E)** A schematic model. All experiments have been repeated three times.

As a co-activator, MKL1 relies on sequence-specific transcription factors be to recruited to target promoters. We have previously shown that NF-κB interacts with MKL1 and brings MKL1 to several of its target promoters [12, 14, 15]. Since HDAC5 dampened promoter occupancies by MKL1, we hypothesized that HDAC5 could interfere with the MKL1-NF-κB interaction leading to reduced MKL1 recruitment. Co-immunoprecipitation assay showed that FLAG-MKL1 interacted with V5-tagged p65 in HEK293 cells only when both proteins were over-expressed. The addition of HDAC5, however, significantly weakened the MKL1-p65 interaction (Figure 6A). In THP-1 cells, silencing of HDAC5 with siRNA allowed a stronger interaction between MKL1 and p65 (Figure 6B). Similar observations were made in RAW264 cells (Supplementary Figure 3).

Next, we performed Re-ChIP experiments to evaluate the impact of HDAC5 on the MKL1-p65 interaction on gene promoters in THP-1 cells. When the cells were exposed to TNF-α, a stronger MKL1-p65 interaction could be detected on the IL-1 promoter, the IL-6 promoter, and TNF-a promoter, but not on the GAPDH promoter (Figure 6C); HDAC5 over-expression disrupted the MKL1-p65 complex on gene promoters. In contrast, HDAC5 depletion enhanced the MKL1-p65 interaction on gene promoters (Figure 6D). Collectively, these data suggest that HDAC5-mediated deacetylation may interfere with the MKL1-p65 interaction.

## DISCUSSION

HDAC5 belongs to the class II lysine deacetylases [23]. Under physiological conditions, HDAC5 is functionally redundant because systemic deletion of HDAC5 in mice does not alter organogenesis or postnatal development [24]. How HDAC5 contributes to pathophysiological processes following embryogenesis remains incompletely understood because most studies so far have focused on the regulatory role HDAC5 plays in cardiac hypertrophy by acting as a co-repressor for MEF2 [25]. Here we provide evidence to show that HDAC5 may play a role in TNF-α induced pro-inflammatory transcription in macrophages by deacetylating MKL1 (Figure 6E).

Unlike its sibling myocardin which is exclusively expressed in the muscle lineage, MKL1 is universally distributed throughout the body [13]. So far, MKL1 has been uniformly considered a promoter of disease progression by programming transcriptional events involved in pathological cardiac hypertrophy [26, 27], colitis [14], sepsis [15], pulmonary hypertension [11], atherosclerosis [28], diabetic nephropathy [29], cirrhosis [30], and carcinogenesis [31]. Therefore, although our current investigation has focused on the regulation of MKL1 activity in macrophages, it is reasonable to conclude that small-molecule compounds that promote MKL1 deacetylation may not only help curb macrophage-dependent inflammation but prove beneficial in the intervention of MKL1-related pathophysiological processes. One caveat that needs to be taken into consideration is the possibility that HDAC5-mediated histone deacetylation may also contribute to the regulation of cellular inflammation. Indeed, Schmeck *et al* have shown that in lung epithelial cells, infection of *Legionella pneumophila* displaces HDAC5 from the IL-8 promoter followed by an increase in histone H4 and histone H3K14 acetylation [32]. These events presumably allow the trans-activation of IL-8 and consequently the development of pneumonia.

Our working model (Figure 6E) centers around MKL1 being the chief substrate for HDAC5 during TNF-α induced pro-inflammatory transcription. An equally plausible scenario would be that NF-κB/p65 is subject to modification by HDAC5. Indeed, several lysine deacetylases have been shown to modulate the acetylation status and hence activity of p65. The class III lysine deacetylase SIRT1, for instance, deacetylates p65 to contain the synthesis of pro-inflammatory mediators [7]. Class I lysine deacetylases HDAC1, HDAC2, and HDAC3 have also been documented to deacetylate p65 to program different pathophysiologically relevant events [33, 34]. It would be of interest to determine whether HDAC5 might directly deacetylate p65.

The *in vivo* relevance of our finding at this point remains an open question. Previous investigations have implicated HDAC5 in the regulation of inflammation-related human diseases. For instance, it has been demonstrated that the massage levels of HDAC5, among other class I and class II HDACs, are down-regulated in the lungs in patients with chronic obstructive pulmonary disease (COPD) [35]. More recently, Angiolilli *et al* have reported that HDAC5 expression is down-regulated in the synovial tissue isolated from patients with rheumatoid arthritis (RA) paralleling up-regulation of pro-inflammatory mediators [36]. Although it is not clear whether macrophages constitute the major source of pro-inflammatory mediators as reported by Ito *et al* [35] and Angiolilli *et al* [36], these findings lend support to a model wherein HDAC5 functions as a key check point reining in cellular inflammation whereas the loss of HDAC5 gives rise to uncontrolled inflammatory response and disease development. Curiously, although a range of HDAC inhibitors have been designed and synthesized, many of which are being used to treat cancers in the clinics, no HDAC5 activator or agonist has been reported. HDAC5 activity itself is regulated by calmodulin-dependent kinase (CaMK)-mediated phosphorylation and nuclear exclusion [37]. CaMK inhibitors have been shown to exert an-inflammatory effects in various cell types although it is not clear whether these effects depend on HDAC5 [38, 39]. Further investigation is warranted to define a more precise role for HDAC5 in the regulation of cellular inflammation in order to aid drug development.

## MATERIALS AND METHODS

### Cell culture and treatment

HEK293 cells and RAW264 cells were maintained in DMEM supplemented with 10% fetal bovine serum (Gibco) [40]. THP-1 cells were maintained in RPMI-1640 DMEM supplemented with 10% FBS. TNF-α (10ng/ml) was purchased from R&D.

### Plasmids, transient transfection, and luciferase assay

FLAG-MKL1 (wild type and acetylation defective), HA-HDAC5, V5-p65, HDAC5 shRNA plasmid, and promoter-luciferase constructs have been described previously [12, 14, 22, 41–44]. Small interfering RNAs were purchased from Dharmacon. Transient transfections were performed with Lipofectamine 2000 or Lipofectamine LTX (Invitrogen). Luciferase activities were assayed 24-48 hours after transfection using a luciferase reporter assay system (Promega). All experiments were performed in triplicate wells and repeated three times.

### Protein extraction, immunoprecipitation and western blot

Whole cell lysates were obtained by re-suspending cell pellets in RIPA buffer (50 mM Tris pH7.4, 150 mM NaCl, 1% Triton X-100) with freshly added protease inhibitor (Roche). Specific antibodies or pre-immune IgGs (P.I.I.) were added to and incubated with cell lysate overnight before being absorbed by Protein A/G-plus Agarose beads. Precipitated immune complex was released by boiling with 1X SDS electrophoresis sample buffer. Alternatively, FLAG-conjugated beads (M2, Sigma, A2220) were added to and incubated with lysates overnight. Precipitated immune complex was eluted with 3X FLAG peptide (Sigma). Western blot analyses were performed with anti-FLAG (Sigma, F3165, 1:3,000), anti-HA (Sigma, H9658, 1:3,000), anti-β-actin (Sigma, A1978, 1:3,000), anti-MKL1, (Santa Cruz, sc32909, 1:1,000), anti-HDAC5 (Abcam, ab1439, 1:2,000), and anti-acetyl lysine (Cell Signaling Tech, 9441, 1:1,000) antibodies. All experiments were repeated at least three times.

### RNA isolation and real-time PCR

RNA was extracted with the RNeasy RNA isolation kit (Qiagen). Reverse transcriptase reactions were performed using a SuperScript First-strand Synthesis System (Invitrogen). Real-time PCR reactions were performed on an ABI Prism 7500 system. Primers and Taqman probes used for real-time reactions were purchased from Applied Biosystems. All experiments were repeated at least three times.

### Chromatin immunoprecipitation (ChIP)

Chromatin Immunoprecipitation (ChIP) assays were performed essentially as described before [45]. In brief, chromatin in control and treated cells were cross-linked with 1% formaldehyde. Cells were incubated in lysis buffer (150 mM NaCl, 25 mM Tris pH 7.5, 1% Triton X-100, 0.1% SDS, 0.5% deoxycholate) supplemented with protease inhibitor tablet and PMSF. DNA was fragmented into ∼500 bp pieces using a Branson 250 sonicator. Aliquots of lysates containing 200 μg of protein were used for each immunoprecipitation reaction anti-FLAG (Sigma, A2220, 1:100), anti-MKL1 (Santa Cruz, sc32909, 1:100), anti-p65 (Cell Signaling, 8242, 1:100), anti-HDAC5 (Santa Cruz, sc133225, 1:100) or pre-immune IgG. Precipitated genomic DNA was amplified by real-time PCR with the previously described primers.

### Immunofluorescence microscopy

FLAG-tagged MKL1 was transfected into HEK293 cells with or without HDAC5 followed by treatment with TNF-α. Cells were fixed with 4% formaldehyde, permeabilized with TBST, blocked with 5% BSA, and incubated with anti-FLAG (Sigma) overnight. After several washes with PBS, cells were incubated with FITC-labeled secondary antibodies (Jackson) for 30 minutes. DAPI (Sigma) was added and incubated with cells for 5 minutes prior to observation. Immunofluorescence was visualized on a co-focal microscope (LSM 710, Zeiss).

### Statistical analysis

One-way ANOVA with post-hoc Scheffe analyses were performed using an SPSS package. P values smaller than.05 were considered statistically significant (^*^).

## SUPPLEMENTARY MATERIALS FIGURES


