# Supplementary material for: HADC5 deacetylates MKL1 to dampen TNF-α induced pro-inflammatory gene transcription in macrophages
Source: Oncotarget. 2017 Oct 9;8(55):94235–46. doi: 10.18632/oncotarget.21670 (PMC5706870; doi:10.18632/oncotarget.21670)
Supplement: Supplementary file 1 [file oncotarget-08-94235-s001.pdf]

HADC5 deacetylates MKL1 to dampen TNF-α induced pro-inflammatory gene transcription in macrophages

SUPPLEMENTARY MATERIALS

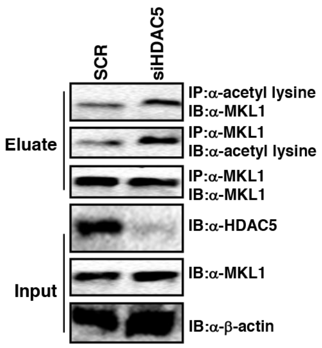

Supplementary Figure 1: RAW264 cells were transfected with siRNA targeting HDAC5 or scrambled siRNA (SCR). Immunoprecipitation assay was performed with indicated antibodies.

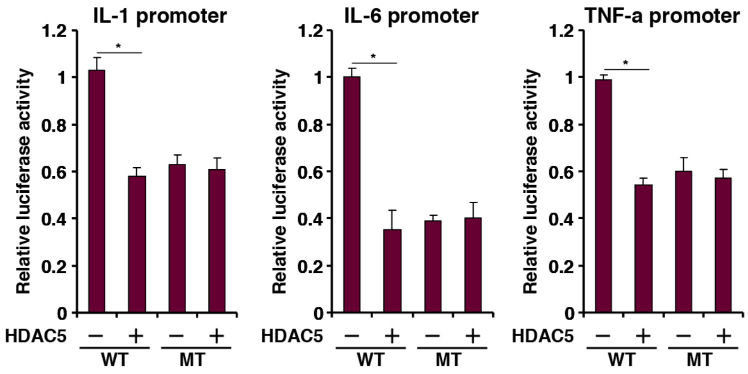

Supplementary Figure 2: Different promoter constructs were transfected into HEK293 cells with wild type (WT) MKL1, acetylation-defective (MT) MKL1, and/or HDAC5 expression construct as indicated. Luciferase activities were normalized by protein concentration and GFP fluorescence.

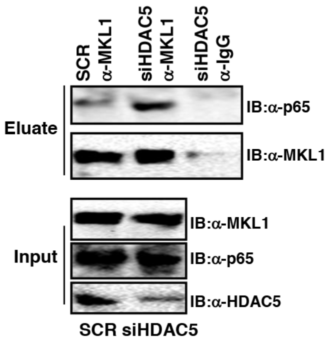

Supplementary Figure 3: RAW264 cells were transfected with siRNA targeting HDAC5 or scrambled siRNA (SCR) followed by treatment with TNF-α (10ng/ml). Immunoprecipitation was performed with anti-MKL1 or IgG.
